# Cesarean section trends from 1992 to 2023 in Estonia among singleton term pregnancies: A registry-based study

**DOI:** 10.18332/ejm/201342

**Published:** 2025-04-01

**Authors:** Kaire Sildver, Piret Veerus, Mika Gissler, Katrin Lang, Heti Pisarev

**Affiliations:** 1 Department of Midwifery, Tallinn Health Care College, Tallinn, Estonia; 2 National Institute for Health Development, Tallinn, Estonia; 3 West-Tallinn Central Hospital, Tallinn, Estonia; 4 Institute of Family Medicine and Public Health, Faculty of Medicine, University of Tartu, Tartu, Estonia; 5 Finnish Institute for Health and Welfare THL, Department of Data and Analytics Helsinki, Finland; 6 Region Stockholm, Academic Primary Health Care Centre, Stockholm, Sweden; 7 Karolinska Institute, Department of Molecular Medicine and Surgery, Stockholm, Sweden

**Keywords:** cesarean section, singleton term pregnancies, time trends, Robson classification, Estonia, registry-based study

## Abstract

**INTRODUCTION:**

The proportion of cesarean section (CS) deliveries has increased worldwide. This study aimed to analyze CS trends in Estonia from 1992 to 2023 in total and according to Robson 1+2 (nulliparous, single cephalic, ≥37 weeks, spontaneous labor, induced or CS before labor) and 5 (previous CS, single, cephalic, ≥37 weeks) criteria.

**METHODS:**

Data of all deliveries (n=446536) in Estonia from 1992 to 2023 were obtained from the Estonian Medical Birth Registry. During the study period, 73960 births ended in CS. Descriptive characteristics of the study population were divided into two periods (1992–2007 increasing trend; 2008–2023 stable trend). Robson 1+2 and 5 sub-groups were analyzed. Joinpoint regression was used to estimate the change in CS trends in Robson groups over time.

**RESULTS:**

The total proportion of CS increased from 6.5% in 1992 to 20.9% in 2007 and remained stable after that. Robson 1+2 proportion increased from 5% to 21% in 2023, and Robson 5 decreased from 73% to 56%. In 2023, R1+R2 combined with R5 accounted for more than half (63%) of all CSs.

**CONCLUSIONS:**

The increase in CS occurred primarily due to the increase in CS rates among nulliparous women with a singleton pregnancy at term. More attention must be given to nulliparous women to prevent CS and maintain vaginal births after CS. To improve the quality of maternity care, it is essential to monitor the indicators of CS based on Robson’s criteria.

## INTRODUCTION

Deliveries can be divided into two types: natural and operative delivery. Operative delivery can, in turn, be divided into two: abdominal operative delivery, which includes the cesarean section (CS), and operative vaginal delivery. The optimal rate of CS has been long debated^1^. Already in 1985, WHO deemed it necessary to determine the optimal proportion of CS to be 10-15%^2^. In 2024, the European Association of Perinatal Medicine and the European Midwives Association highlighted CS rates as a significant issue, recommending that national CS rates should be within the range 15–20%^3^. The WHO guideline states that CS is a life-saving operation only when medically indicated^4^. The rates of CS vary in Europe largely. CS rates in Europe (with a median rate of 27.0% in 2015 and 26.0% in 2019) ranged from 16.1% in Iceland to 56.9% in Cyprus in 2015, and from 16.4% in Norway to 53.1% in Cyprus in 2019^5,6^. The proportion of CS in Nordic countries is lower compared to the rest of Europe^7^.

Robson^8^ proposed using the Ten-Group Classification System (TGCS), which applies to all women, not just those who deliver by CS. Robson’s system is based on obstetric parameters: parity, previous CS, gestational age, the onset of labor, fetal presentation, and the number of fetuses^8^. The WHO Statement proposed using the TGCS as the global standard for assessing, monitoring and comparing CS rates within healthcare facilities over time and between facilities in 2015^4^. The International Federation of Gynecology and Obstetrics (FIGO) encouraged the uniform collection of perinatal data using the TGCS in 2016^9^.

In Europe, several countries have used the TGCS to research CS time trends. Each country can monitor the CS rate separately based on Robson’s classification criteria (WHO Robson Classification Implementation manual) and Robson guideline target values^10^. Based on the data from 28 countries in the Euro-Peristat study, the results show that from 2015 to 2019, in countries where CS rates increased by more than >1%, this was primarily seen in groups R1 (nulliparous, single, cephalic, ≥37 weeks, spontaneous labor), R2 (nulliparous, single, cephalic, ≥37 weeks, induced or CS before labor), R4 (multiparous, single cephalic, ≥37 weeks, induced or CS before labor), and R10 (preterm, cephalic, singletons)^11^. A population-based registry study from 2000 to 2011 in Nordic countries found that CS total rates increased the most in R1+R2 and R5 (previous CS, single, cephalic, ≥37 weeks) groups^7^. A European study recommends that each country conduct a survey based on the Robson classification to identify the proportion of CS in each country and, if necessary, to plan interventions^12^.

In Estonia, the CS rate was 6.3% in 1992^13^, and by 2023 had risen to 21%^14^. A CS is performed in Estonia only for medical reasons. Health insurance is guaranteed to be free of charge to all pregnant women. In Estonia, pregnancy can be monitored in both private and public clinics. However, childbirth is only possible in a public maternity hospital or, in the case of a low-risk pregnancy, at home. The proportion of home deliveries is very low. The pregnancy is generally monitored by a midwife (an average of 10 visits, two of which are with a gynecologist), who refers the woman for examinations and meets every month. Throughout the pregnancy, midwives prepare women for childbirth. A midwife can consult with other medical specialists and, if necessary, refer the woman or family to specialist appointments^15^.

The current article aims to provide an overview of total CS trends in Estonia from 1992 to 2023, with a particular focus on CS according to Robson 1+2 and 5 criteria. To analyze changes over time, the background characteristics were compared in two time periods: 1992–2006 and 2007–2023.

## METHODS

All deliveries in Estonia in the years 1992–2023 were included in this study. The data were obtained from the Estonian Medical Birth Registry and included the following variables: mother’s age; number of previous deliveries; pregnancy risk factors: previous CS; gestational age; puerperal and postnatal diagnoses: breech presentation, other atypical fetal condition; mode of delivery: vaginal delivery, planned CS, other cesarean section; other operations on delivery: induction of labor; number of children; and birth weight.

**Figure 1:**
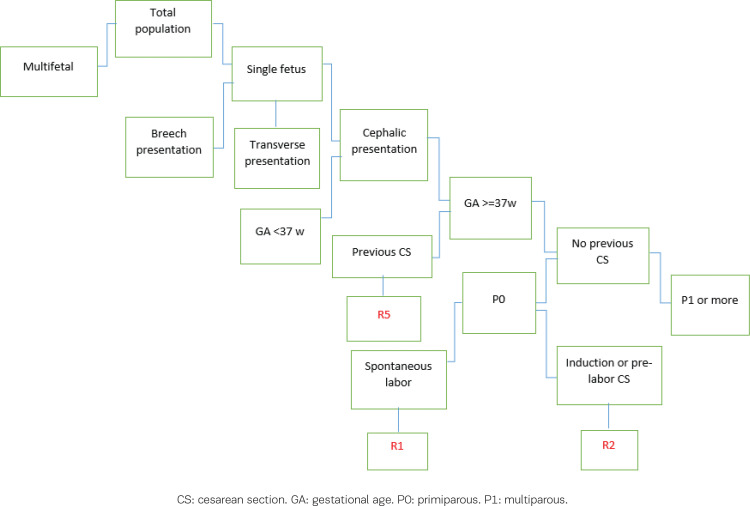
Classification process of women into the Robson groups R1, R2, and R5

Figure 1 shows deliveries in Robson groups categorized as follows R1 (Robson 1): nulliparous, single, cephalic, ≥37 weeks, spontaneous labor; R2 (Robson 2): nulliparous, single, cephalic, ≥37 weeks, induced or CS before labor; R5 (Robson 5): previous CS, single, cephalic, ≥37 weeks^8^.

The Robson classification should be considered as a common starting point. However, some groups may require further subdivision, or it may be necessary to combine groups. The WHO Robson Classification Implementation Manual states that the larger the ratio between the sizes of Groups R1 and R2, the more likely it is that the CS rate in the group will not fall within the target value. However, if R1 and R2 are combined, the CS rate may still remain within the recommended range^10^.

### Statistical analysis

The total number of births in the 1992–2023 period was 446536. We examined maternal characteristics and clinical and obstetrical factors. Data were divided into two periods (1992–2007 with increasing trend; 2008–2023 with stable trend) with a focus on the following indicators: parity, nulliparous; cesarean section; gestational age, ≥37 weeks; onset of labor, induction; previous CS; presentation, cephalic; singleton pregnancy; maternal age; and birthweight. Statistical differences between the groups used Fisher’s exact test, and p<0.05 was considered statistically significant.

In this study, the R1 and R2 groups are combined. The Robson groups 1+2 and 5 have the most significant effect on the overall proportion of CS; therefore, this study focuses on them. The CS rates were calculated by dividing the number of CSs by the total number of deliveries in R1+2 and R5 groups and expressing it as a percentage. During the study period (1992–2023), 73960 births ended in CS.

The method of joinpoint regression has been used to assess changes in time series data and helps to achieve an accurate and comprehensive understanding of population dynamics^16^. Joinpoint regression was used to estimate the change in R1+2 and R5 groups’ CS trends over time, and the results are presented in annual percentage change (APC). Statistical software used was Stata/IC 14 (StataCorp LLC, College Station, TX, USA) and the Joinpoint Regression Program 5.0.2.

### Ethical approval

The study was approved by the Tallinn Medical Research Ethics Committee, National Institute for Health Development. The National Institute for Health Development gave permission to use the Estonian anonymized registry data.

## RESULTS

In the period 1992–2023, 73960 of 446536 births in Estonia ended in CS (16.6%). Table 1 shows that the average age of mothers in Estonia has risen steadily over the last 31 years. In 1992–2006, the average age of mothers was 26.9 years; in 2007–2023, it increased by 3 years (to 29.9 years). The number of singleton pregnancies decreased from 97.9% to 96.8%. While in 1992–2006, 49.3% of all births were by the mothers delivering their first baby, by 2007–2023, this indicator decreased to 43.6%. The proportion of induced labor increased significantly over time; in 1992–2006, it was 7.0% of all births, and by 2007–2023 it had risen to 16.1%. The percentage of CS has increased from 13.4% to 19.3% in the period from 1992–2006 to 2007–2023. As a result, the number of deliveries with a previous CS also increased significantly. In 1992–2006, 3.5% of women had a history of previous CS; by 2007–2023, this proportion had risen to 10.1%. The proportion of full-term pregnancies (gestational age, ≥37 weeks) remained unchanged (89–90%) throughout the study period. The average birthweight of children also increased by 33 g (3478 to 3511 g). All descriptive characteristics of the study population 1992–2006 and 2007–2023 have a statistically significant difference, excluding cephalic presentation with no difference in the two time periods.

**Table 1:** Characteristics of the study population in Estonia, 1992–2006 and 2007–2023

*Characteristics*	*1992–2006 n (%)*	*2007–2023 n (%)*	*p*
Births, n	206897	239639	<0.001
Maternal age (years), mean (SD)	26.9 (5.6)	29.9 (5.6)	<0.001
Parity, nulliparous	101945 (49.3)	104452 (43.6)	<0.001
Singleton pregnancy	202498 (97.9)	232041 (96.8)	<0.001
Presentation, cephalic	198019 (95.7)	229099 (95.6)	0.11
Onset of labor, induced	14536 (7.0)	38630 (16.1)	<0.001
Cesarean section (CS)	27758 (13.4)	46202 (19.3)	<0.001
Multiparous, previous CS	7250 (3.5)	24148 (10.1)	<0.001
Gestational age, ≥37 weeks	184173 (89.0)	216656 (90.4)	<0.001
Birth weight (g), mean (SD)	3478 (587.5)	3511 (590.7)	<0.001

Statistically significant differences p<0.05.

**Figure 2:**
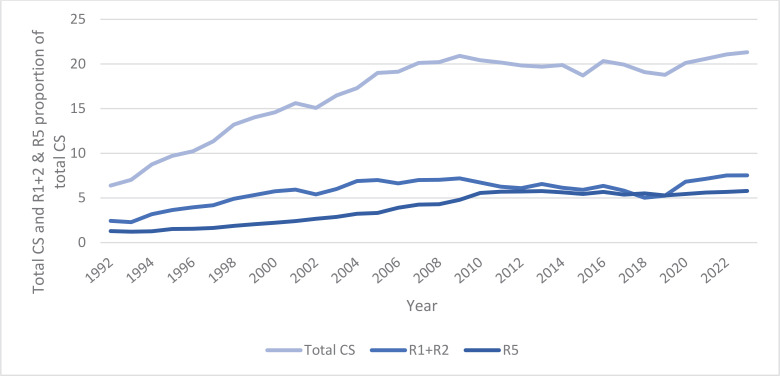
Total CS time trend, and R1+R2 and R5 proportion of total CS rates in Estonia, 1992–2023

Figure 2 shows that in 1992, the total percentage of CS was 6.5%. The total percentage of CS was extended on the rise, but since 2007, it has remained stable between 19% and 21%. CS in the R1+2 group showed an upward trend until 1999, accounting for 5–7% of total CSs (about 20%). The proportion of CS in the R5 group increased until 2010; since then, it has remained within the 5% range of the total proportion of CS. In 2023, CS accounted for 21.3% of all births, with CS in the R1+2 group 7.5% and those in the R5 group accounting for 5.7%. In 2023, R1+2 combined with R5 accounted for more than half of all CSs.

**Figure 3:**
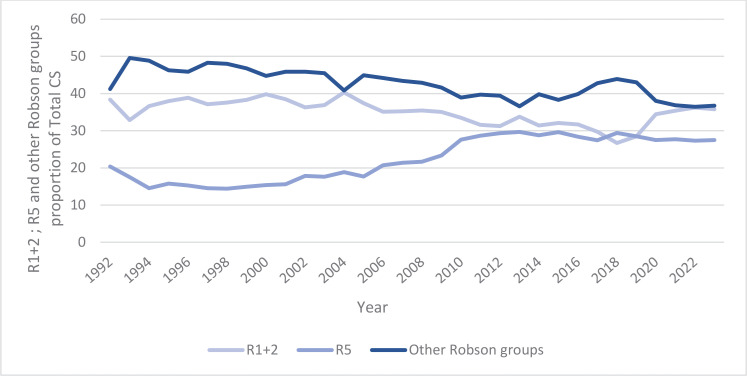
R1+R2, R5 and other Robson groups time trends and proportion of the total CS in Estonia, 1992–2023

Figure 3 shows that the other Robson groups constituted most of all CSs for a long time. The proportion of the R1+2 group has been lower than that of the other Robson groups throughout the period (except in 2004), but since 2020, they have been similar. In 2023, the R1+2 group accounted for 35%, while the other Robson groups accounted for 36%. The R5 group has been on a long-term upward trend, stabilizing around 27% in recent years.

**Figure 4:**
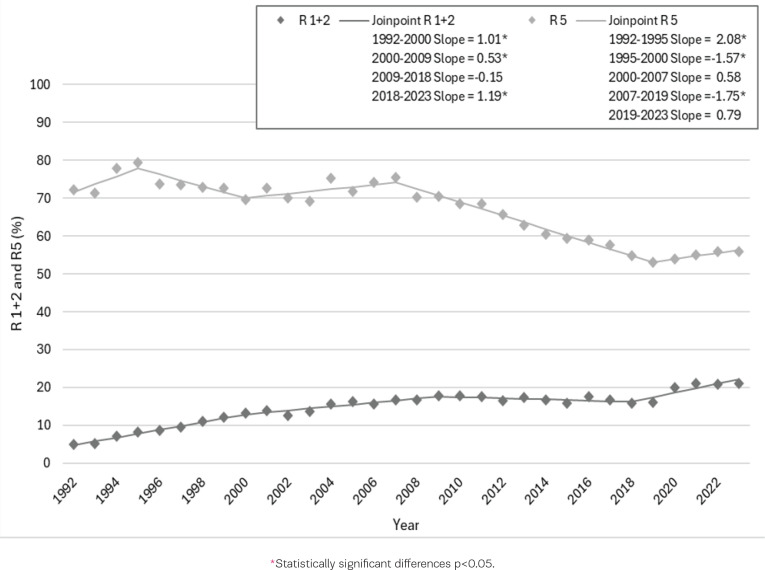
Time trends and proportion of R1+R2 and R5 from all births and annual percent change (APC) in Estonia, 1992–2023

Figure 4 shows the percentage of CS birth trends in the Robson groups 1+2 and 5 and the annual percent change (APC). Robson group 1+2 (nulliparous, single cephalic, ≥37 weeks, spontaneous labor, induced or CS before labor), the percentage of CS has increased from 5% to 21% in the total period. According to Joinpoint regression, the increase in R1+2 can be divided into four periods. In the first period between 1992 and 2000, the share of R1+2 increased by 1.01% per year. From 2000 to 2009, the increase slowed down in the next period but continued to grow at 0.53% per year. Then, the growth stopped until 2018, when it turned upward again. The changes in the first two time periods and the last period are statistically significant. For the Robson group 5 (previous CS, single, cephalic, ≥37 weeks), more than 70% of births in 1992–2009 ended in CS, reaching up to 80%, but since 2007 the percentage has been steadily falling and reached 56% in 2023. Changes in R5 can be divided into five periods, and a significant change in the proportion of R5 has occurred in three periods. The share of R5 increased by 2.08% per year in the period 1992–1995, and in the period 1995–2000 decreased by 1.57%. After that, the decrease slowed and turned to increase, but from 2007 to 2019, the annual decline remained at 1.75%, and in the last period, it stagnated again.

## DISCUSSION

In this large study covering more than 30 years (1992–2023), the number of births in Estonia decreased significantly, and at the same time, the proportion of total CSs increased from 6.5% to 21.3%. The percentage of CS births in nulliparous women with a singleton cephalic pregnancy at term (R1+2) increased from 1992–2023 from 5% to 21%. More than 70% of births in multiparous previous CS women with a singleton cephalic pregnancy at term (R5) ended before 2009 in CS, but it has been falling and reached 56% in 2023. More than half of the total CSs (63%) in 2023 is accounted for by cesarean sections from groups R1+2 and R5.

The reason for the present high share of CS in several countries is multifactorial. It is based on the healthcare system (guidelines, legal fears), healthcare providers, financial interests, women’s individuality, society, fashion, and the media^4^. Today, Estonia is one of the safest countries in the world for giving birth, for mothers and children^5^. This change has been achieved in the last 30 years, and the maternity care indicators are excellent. Following public health policy reforms in 1991–1994, establishing a solidarity-based Health Insurance Fund^17^, and regularly monitoring obstetric indicators^18^, the Estonian Gynecologists’ Society routinely assesses scientific advancements and introduces updated practice guidelines^19^. Compliance with these guidelines aims to balance the potential negative impacts of evolving medical trends in maternity care while addressing economic and social influences on childbirth decisions. Enhanced access to perinatal and obstetric care in Estonia has resulted in a significant decline in perinatal mortality. Estonia’s perinatal mortality rate (per 1000 live births) has steadily declined since 1992, when it was 19.8/1000^13^ , and in 2023 decreased to 3.4/1000^14^.

Benchmarks are set for Robson’s groups to determine target values. Separately, Robson 1 (nulliparous, single, cephalic, ≥37 weeks, spontaneous labor) should be less than 10%, and Robson 2 (nulliparous, single, cephalic, ≥37 weeks, induced or CS before labor) should be 20–35%^10^. The share of the Robson group 2 is increasing, as is the share of induced births in Estonia and elsewhere worldwide. In developed countries, about 20–30% of births are induced. In 2023 in Estonia, 29.4% of births were induced. However, after combining R1 and R2, the value is more likely to be within the target value of 10% to 35%^10^.

In 2013, the Advisory Council for Treatment Quality Indicators was established in collaboration with the Estonian Health Insurance Fund and the University of Tartu. This council set principles for selecting indicators that reflect treatment quality, with professional societies proposing indicators evaluated against these principles. As a result, five key obstetric care indicators have been developed in Estonia. Among them are the rate of CS in primiparous women with singleton pregnancies and cephalic presentations (Robson 1+2) and the rate of CS in multiparous women with a history of cesarean delivery and cephalic singleton pregnancies (Robson 5). These indicators help monitor treatment quality and resource utilization, ensuring the healthcare system operates efficiently^18^.

The percentage of CS births in Estonia in 2016 in Robson groups 1+2 was 19.3%. In France, in 2016, it was 17.5%^20^. A Nordic study found that all Nordic countries had a lower R1+2 CS rate in 2009–2011 than in Estonia: Finland (16.8%), Sweden (14.3%), Denmark (15.7%), and Norway (13.8%)^7^. Nordic studies have suggested that increased R1 and R2 groups lead to a higher overall CS rate^7^. To lower the total CS rate, it is essential to reduce cesarean sections in nulliparous women with single, cephalic, full-term pregnancies (R1 and R2)^21^.

The target value of Robson 5 (previous CS, single, cephalic, ≥37 weeks) should be 50–60%^10^. In Estonia, the share of CS in the R5 group is large and has reached 80%, but since 2007, the share of CS has been steadily declining. This may be because, in the 20th century, it was expected that if a woman had given birth to one child in CS, the following births were also in CS^22^. However, now the need for a CS is increasingly questioned, and even births by vaginal delivery occur even after an earlier CS^22^. A Euro-Peristat study based on 2015 data showed that Estonia’s indicators in Robson Group 5 were similar to those of the Nordic countries, staying within the recommended limit with a CS rate below 60%. However, there are many European countries where the CS rate in Group 5 is higher, such as Slovenia, Germany, Malta, and Belgium, and it exceeds 80% in Latvia, Italy, and Cyprus^23^. In Estonia, vaginal delivery after birth is strongly encouraged, and CS on demand is not allowed. By 2023, the share of CS in the Robson group 5 decreased to 56% in Estonia. In the future, CS trends in all Robson groups should be monitored.

### Strengths and limitations

A strength of our study was the use of the Estonian Medical Birth Registry data covering more than three decades. Data on all 446536 births for the period covered the whole of Estonia. The Estonian Medical Birth Registry was established in 1991 to collect data on the perinatal period, and the data collection started on 1 January 1992^24^. The Finnish Medical Birth Registry was used as a model when creating the Estonian one^13^. The data were collected on the birth card, which was changed in 1994, 1998, and 2020. However, these changes did not influence the composition of the analyzed data. The quality of the Estonian Medical Birth Register data was studied from 1997: registry data were mainly comparable with birth records^25^, and we used only highly quality variables.

The limitation of this study is that various factors can affect the proportion of CS deliveries, including high birth weight^26^, maternal characteristics such as BMI^27^, age^28^, and chronic diseases^29^. Additionally, prior studies have shown that the use of epidural anesthesia significantly impacts the number of cesarean deliveries^30^. The effects of these factors on CS rates were not analyzed separately. Another limitation is that R1 and R2 subgroups were not analyzed separately in this study.

## CONCLUSIONS

To improve the quality of maternity care, it is important to monitor the quality-of-care indicators and CS trends, preferably based on Robson’s criteria, which allow an objective comparison over time and between countries. More attention must be given, and an evidence-based approach must be applied to nulliparous women to prevent unnecessary CS and to promote more vaginal births after CS. The availability of good-quality registry data is essential.

## Data Availability

The data supporting this research cannot be made available for privacy reasons. The data supporting this study’s findings can be accessed through the Estonia National Institute for Health Development. Register data are protected by confidentiality rules. Researchers can only access the data after a special review that includes ethical approval from the authorities controlling the data and regional Ethics Committees.
